# Induction of Fatigue by Specific Anthracycline Cancer Drugs through Disruption of the Circadian Pacemaker

**DOI:** 10.3390/cancers14102421

**Published:** 2022-05-13

**Authors:** Yumeng Wang, Sabina Y. van der Zanden, Suzanne van Leerdam, Mayke M. H. Tersteeg, Anneke Kastelein, Stephan Michel, Jacques Neefjes, Johanna H. Meijer, Tom Deboer

**Affiliations:** 1 Department of Cell and Chemical Biology, Leiden University Medical Center, 2333 ZC Leiden, The Netherlands; y.wang.mcb@lumc.nl (Y.W.); suzannevanleerdam@hotmail.com (S.v.L.); m.m.h.tersteeg@lumc.nl (M.M.H.T.); a.kastelein@lumc.nl (A.K.); s.h.michel@lumc.nl (S.M.); j.h.meijer@lumc.nl (J.H.M.); 2 Department of Cell and Chemical Biology, ONCODE Institute, Leiden University Medical Center, 2333 ZC Leiden, The Netherlands; s.y.van_der_zanden@lumc.nl (S.Y.v.d.Z.); j.j.c.neefjes@lumc.nl (J.N.)

**Keywords:** cancer-related fatigue, anthracyclines, quality of life, circadian, sleep

## Abstract

**Simple Summary:**

Cancer-related fatigue (CRF) is a devastating side effect of cancer treatment, affecting the quality of life of many patients for years after treatment. This long-term side effect often results in loss of social functioning and even job loss. The cause of CRF is unknown, and consequently, CRF is often considered a ‘psychological problem’, much to the frustration of the patients. Here, we show in an animal model that the severity of CRF depends on the working mechanism of the treatment. In addition, the data show that the CRF is probably caused by a dysfunctioning circadian clock and thus has a physiological basis, as this effect depends on the anticancer drug. Therefore, the findings may have implications for the selection of chemotherapy and thus strongly improve the quality of life of future cancer survivors.

**Abstract:**

Cancer-related fatigue (CRF) is the most devastating long-term side effect of many cancer survivors that confounds the quality of life for months to years after treatment. However, the cause of CRF is poorly understood. As a result, cancer survivors, at best, receive psychological support. Chemotherapy has been shown to increase the risk of CRF. Here, we study therapy-induced fatigue in a non-tumor-bearing mouse model with three different topoisomerase II-poisoning cancer drugs. These drugs either induce DNA damage and/or chromatin damage. Shortly before and several weeks after treatment, running wheel activity and electroencephalographic sleep were recorded. We show that doxorubicin, combining DNA damage with chromatin damage, unlike aclarubicin or etoposide, induces sustained CRF in this model. Surprisingly, this was not related to changes in sleep. In contrast, our data indicate that the therapy-induced CRF is associated with a disrupted circadian clock. The data suggest that CRF is probably a circadian clock disorder that influences the quality of waking and that the development of CRF depends on the type of chemotherapy provided. These findings could have implications for selecting and improving chemotherapy for the treatment of cancer in order to prevent the development of CRF.

## 1. Introduction

Cancer-related fatigue (CRF) is a complex and debilitating side effect of cancer treatment. According to the American Cancer Society website: ‘People with cancer might describe it [CRF] as feeling very weak, listless, drained, or “washed out” that may decrease for a while but then comes back. Some may feel too tired to eat, walk to the bathroom, or even use the TV remote. It can be hard to think or move. Rest might help for a short time but does not make it go away, and just a little activity can be exhausting. For some people with cancer, this kind of fatigue causes more distress than pain, nausea, vomiting, or depression’ (https://www.cancer.org/treatment/treatments-and-side-effects/physical-side-effects/fatigue/what-is-cancer-related-fatigue.html (accessed on 1 April 2022)).

It affects more than 60% of cancer patients and survivors, but its etiology is unknown [1]. Persistent CRF has a serious effect on the quality of life of cancer survivors and can result in conditions where patients are too tired to go to work, socialize, or perform normal daily activities [2,3]. Many cancer patients report an increment in fatigue in the course of treatment with either chemotherapy, radiotherapy, or surgery [4]. Therapy-related CRF can persist for weeks, months, and up to many years following the finalization of treatment but varies between patients and, potentially, treatment modalities. Unfortunately, the relationship between the type of cancer treatment and CRF, as well as the molecular basis for CRF, remains unknown.

Although the cause of CRF is poorly understood, and the relative contribution of tumor type, cancer stage, and individual health status is unknown [5], converging evidence points to a major role of cytostatic drugs, including doxorubicin, in the induction of CRF [6]. Multiple mechanisms underlying the development of therapy-induced CRF have been proposed, such as inflammation, ROS formation, mitochondrial dysfunction, and central nervous system disorder [7,8,9]. Doxorubicin and its anthracycline drug family members are a cornerstone in cancer therapy, either as a single treatment or in a combination regimen, in the treatment of many solid and hematological tumors. For a long time, it was thought that the classical anti-tumor activity for these anthracycline drugs was the inhibition or poisoning of topoisomerase II [10,11]. Topoisomerase II is an enzyme critical in the relaxation of tension in the DNA, condensation of chromatin, and decatenation of DNA. To do so, topoisomerase II introduces a DNA double-strand break (DSB), followed by DNA relaxation and re-ligation. Most anthracyclines, including doxorubicin and the structurally unrelated inhibitor etoposide, interfere with the topoisomerase-DNA interface to prevent re-ligation resulting in DNA DSBs [12]. Rapidly dividing cells, including most tumor cells, should be more sensitive to DNA DSBs than normal quiescent cells, which may explain the therapeutic window for these drugs. More recently, it became clear that anthracyclines, unlike etoposide, also induce chromatin damage as the result of histone evictions [13,14]. Studying the chemical structure of these drugs showed that chromatin damage is likely the major cause of the anticancer activity of most anthracycline drugs [15,16]. Of note, the DNA-damaging activity may conspire with chromatin damage to cause a number of serious side effects associated with anthracycline treatment, including dose-dependent cardiotoxicity and therapy-related malignant neoplasms [15].

We set out to study the putative mechanism underlying the development of therapy-induced CRF by evaluating the effect of three topoisomerase II inhibitors (doxorubicin, etoposide, and aclarubicin) on wheel-running and sleep–wake behavior in mice, which have been used previously as an overt measure of fatigue [17,18]. Wheel running in mice is voluntary, motivated behavior [19] and reflects natural activity levels of mice in the wild [20]; it is, therefore, a suitable outcome measure for a fatigue mouse model. Wheel-running activity has been recently developed as a model to measure CRF in mice, but so far, it has only been used in a limited number of studies evaluating a limited number of therapies [17,21,22,23,24].

We observed that healthy mice treated with doxorubicin showed decreased wheel-running activity, indicating fatigue-like symptoms. Surprisingly, these persistent fatigue symptoms were not associated with disrupted sleep–wake behavior. Instead, we found therapy-induced fatigue likely to be associated with disruption of the central circadian pacemaker. Our study thus suggests that CRF is not a sleep but rather a wake quality issue related to disruption of the circadian clock. This is likely caused by combined DNA- and chromatin damage activity since treatment with the topoisomerase II inhibitor family members aclarubicin (only inducing chromatin damage) or etoposide (only inducing DNA damage) failed to induce altered wheel-running behavior or affect the circadian clock. We thus provide data showing that CRF is dependent on the type of chemotherapy. CRF is then not only a psychological phenomenon but also has a physical cause, a finding that will be highly appreciated by cancer survivors. These data are important to improve the quality of life of cancer survivors and motivate new treatment options, as fatigue is experienced as the most devastating side effect of chemotherapies.

## 2. Materials and Methods

### 2.1. Reagents

Doxorubicin and etoposide were obtained from Pharmachemie (Haarlem, The Netherlands). Aclarubicin (sc-200160) was purchased from Santa Cruz Biotechnology (Santa Cruz, CA, USA), dissolved in dimethylsulfoxide at 5 mg/mL concentration, aliquoted, and stored at −20 °C for further use.

### 2.2. Cell Culture

Endogenous tagged scarlet-H2B cells [15] were cultured in Dulbecco’s Modified Eagle’s medium (DMEM) supplemented with 8% FCS. MelJuSo cells stably expressing PAGFP-H2A were maintained in IMDM supplemented with 8% FCS and G-418, as described [13]. All cell lines were maintained in a humidified atmosphere of 5% CO_2_ at 37 °C and regularly tested for the absence of mycoplasma.

### 2.3. Fractionation Assay

Cells were treated as indicated and washed and lysed directly in lysis buffer (50 mM Tris-Hcl pH 8.0, 150 mM NaCl, 5 mM MgCl_2_, 0.5% NP40, 2.5% glycerol supplemented with protease inhibitors, 10 mM NMM, and 10 µM MG132). Subsequently, cell lysates were collected, vortexed, and incubated for 10 min on ice. To collect the cytosolic fraction, samples were centrifuged for 10 min, 15,000 g, at 4 °C. Both nuclear (pellet) and cytosolic (supernatant) fractions were washed and prepared for Western blot analysis.

### 2.4. Western Blot

Upon treatment, as indicated, cells were washed extensively to remove drugs. Cells were collected and lysed directly in an SDS-sample buffer (2% SDS, 10% glycerol, 5% β-mercaptoethanol, 60 mM Tris-HCl pH 6.8, and 0.01% bromophenol blue). Lysates were resolved by SDS/polyacrylamide gel electrophoresis followed by Western blotting. Primary antibodies used for blotting: γH2AX (1:1000, 05-036, Millipore), β-actin (1:10,000, A5441, Sigma), RFP (1:2000, 6G6, Chromotek, Planegg-Martinsried, Germany), Lamin A/C (1:500, sc-20681, Santa Cruz Biotechnolgy, Santa Cruz, CA, USA), Calnexin (1:1000, C5C9, Cell signaling Technology, Danvers, MA, USA). Images were quantified with ImageJ.

### 2.5. Microscopy

For cytosolic H2B detection, endogenous tagged scarlet-H2B cells were seeded on coverslips. Upon treatment with 10 µM of the indicated drugs for 1 h, cells were fixed in formaldehyde (FA) 4%, permeabilized with 0.1% Triton, and stained with anti-RFP (1:100, 6G6, Chomotek, Planegg-Martinsried, Germany), goat-anti-mouse-Alexa Fluor 488 (1:400, Thermo Fisher Scientific, Waltham, MA, USA) and Alexa Fluor 647 phalloidin (1:125, A22287, Thermo fisher Scientific). Cells stably expressing PAGFP-H2A were used for histone eviction experiments. Photoactivation and time-lapse confocal imaging were performed as described [13,15], and loss of fluorescence from the photoactivated region after different treatments was quantified. Cells were analyzed by a Leica SP8 confocal microscope system with 63× lens. For live-cell imaging, a microscope equipped with a climate chamber was used. Quantification was done using ImageJ software.

### 2.6. Animals

Male C57BL/6J mice (10 weeks old; obtained from Envigo, Horst, The Netherlands) were group-housed in the animal facility of the Leiden University Medical Center (LUMC, Leiden, The Netherlands) under controlled conditions (12:12 h light–dark cycle; lights on at 10:00) with food and water ad libitum in a temperature-controlled room (21–24 °C). They were placed in the recording cabinets for two weeks to familiarize themselves with the new environment and entrain to the new light–dark schedule. Experiments were performed according to institutional and national guidelines and approved by the Animal Ethics Committee of the LUMC (Leiden, The Netherlands).

### 2.7. Behavioral Activity

Running wheel (diameter: 24 cm) activity and passive infrared (PIR) cage activity were recorded with Clocklab data collection software (Actimetrics, Wilmette, IL, USA) and were stored in 1-min bins [25]. Group housed mice (10 weeks old) were provided with a running wheel for two weeks. Subsequently, at 12 weeks of age, mice were individually housed with a PIR sensor and running wheel in a 12:12 h light–dark cycle. Baseline (BL) recordings for the running wheel and PIR activity were done for two weeks, followed by cytostatic treatment. During the treatment and recovery period, as well as during EEG/EMG recordings, only PIR activity was recorded.

### 2.8. EEG/EMG Surgery

Mice were anesthetized (Ketamine 100 mg/kg; Xylazine 10 mg/kg; Atropine 1 mg/kg) and implanted with EEG recording screws (placed above the somatosensory cortex and cerebellum) and electromyogram (EMG) electrodes (placed on the neck muscle) (Plastics One Roanoke, VA, USA), as described previously [25,26]. The wire branches of all electrodes were set in a plastic pedestal (Plastics One, Roanoke, VA, USA) and fixed to the skull with dental cement. Buprenorphine (0.1 mg/kg temgesic) was administered to provide post-surgery analgesia along with a heat lamp until the mice were able to move. Subsequently, the mice were allowed to recover for 7 days.

### 2.9. EEG/EMG Recording

The baseline recordings of RW and PIR were followed by EEG and EMG recordings (Data Sciences International). Animals (control n = 9, doxorubicin n = 8, aclarubicin n = 8, etoposide n = 7) were placed into experimental chambers, connected through a flexible cable and a counterbalanced swivel system to the recording setup. Conditions in the experimental chamber were similar to the home cage, including food and water availability. Before starting the experiment, the animals were allowed to adjust to the experimental conditions for 3 days. Subsequently, a baseline day (24 h) prior to treatment was recorded as previously described [25,26,27], starting at lights on. A post-treatment day (24 h) was recorded 21 days after the last treatment with cytostatic was finished.

### 2.10. Cytostatic Treatment

Mice (n = 7–9 per group) were treated as indicated (i.p.) 4 times (day 1, 2, 9 and 10) over a 10-day period with 3.75 mg/kg doxorubicin, 3.75 mg/kg aclarubicin, 11.25 mg/kg etoposide or 3.75 mL/kg saline with 4% DMSO one hour after light on. The repeated treatment with doxorubicin over 10 days added up to a total of 15 mg/kg as applied previously in [17] in a fatigue study on doxorubicin. Aclarubicin and etoposide were given in dosages relative to doxorubicin based on our previous work in mice [15].

### 2.11. Data and Statistical Analysis

For the in vitro experiments, each sample was assayed in biological triplicate unless stated otherwise. All error bars denote SD. Statistical analyses were performed using Prism 8 software (Graphpad Inc. La Jolla, CA, USA). Western blot and confocal data were quantified using ImageJ software. Significance is represented on the graphs as follow: ns, not significant; * *p* < 0.05; ** *p* < 0.01; *** *p* < 0.001; **** *p* < 0.0001. No statistical methods were used to predetermine sample size. Running-wheel and PIR data were recorded by ClockLab software (Actimetrics, Wilmette, IL, USA) and further analyzed in 1-h values. The strength of the circadian clock and period of free running, wheel running, and cage activity were derived from F-periodogram analysis as previously described [25,28]. Running distance was calculated by counting the number of turns of the wheel. Activity onset was identified through ClockLab software as the activity level that exceeds exactly 20% of all non-zero counts for a 5-h period of inactivity followed by at least a 5-h period of activity. When necessary, onset points were edited manually. Vigilance states were manually scored offline using 4 s epochs and were subdivided into waking, NREM, and REM sleep. EEG power spectral analysis was done by a fast Fourier transform within the range of 0.25–25 Hz, as described previously [27]. Statistical analyses were performed using Prism 8 software (Graphpad Inc., La Jolla, CA, USA) and IBM SPSS Amos 25.0.0 (SPSS Inc., Chicago, IL, USA). Two-way ANOVA with Bonferroni multiple comparisons was used to compare the effect of different cytostatic across time. One-way ANOVA followed by an independent *t*-test was used to compare the strength of the circadian clock. Mann–Whitney U test was used to compare the cumulative running distance among different groups. Independent sample *t*-tests were used to compare Qp and tau between exercise and no-exercise groups.

## 3. Results

### 3.1. Doxorubicin-Treated Mice Develop Fatigue-Like Symptoms

**Figure 1:**
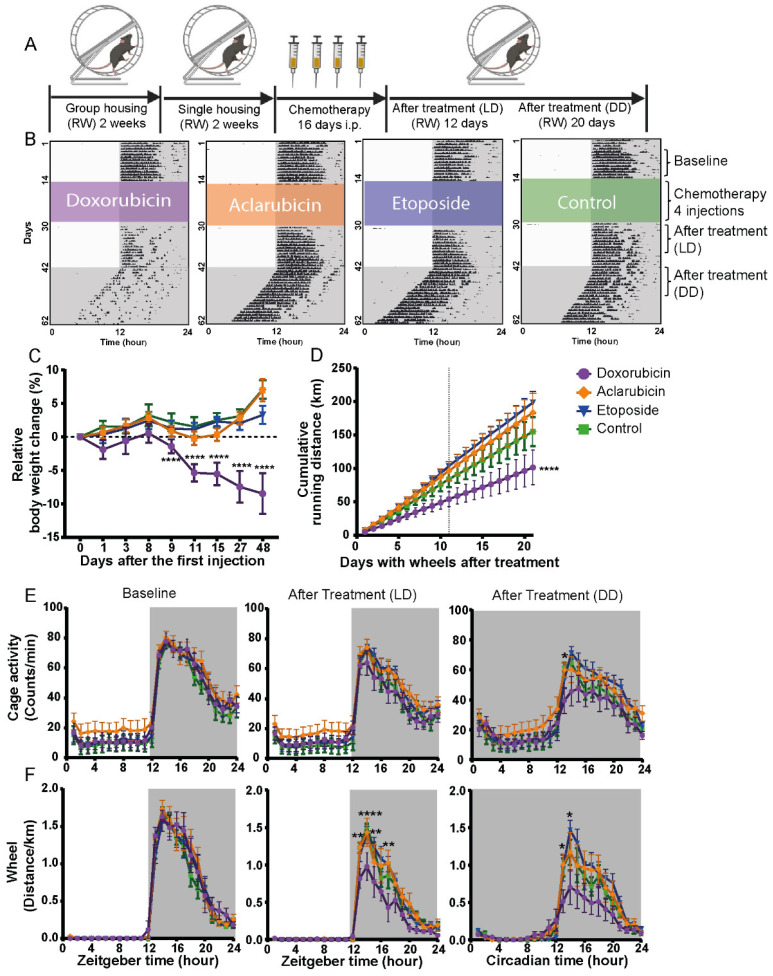
Doxorubicin treatment affects wheel-running activity under light-dark and constant dark conditions. (**A**) Experimental design of the rest-activity behavior study. RW—running wheel; LD—light-dark; DD—constant dark. (**B**) Representative single plotted actograms of wheel-running activity under different chemotherapy treatments during the whole experiment. Black areas indicate wheel activity. Colored areas indicate the treatment time with different cytostatic drugs. (**C**) Relative body weight changes after chemotherapy of the four groups (**** *p* < 0.0001 two-way ANOVA with Bonferroni multiple comparisons, doxorubicin n = 8, all other treatments n = 9 per group). (**D**) Cumulative running distance during the DD phase (active phase) across 21 days, starting 7 days after the last injection. Vertical line indicated the mice were under DD condition (**** *p* < 0.0001, Mann–Whitney U test). (**E**) Average cage activity PIR counts/min over 10 days under baseline condition (left), treatment LD condition (middle), and treatment DD condition (right). (**F**) Average running distance/h over 10 days under baseline condition (left), treatment LD condition (middle), and treatment DD condition (right) (* *p* < 0.05, ** *p* < 0.01, **** *p* < 0.0001 doxorubicin compared to control group, two-way ANOVA with Bonferroni multiple comparisons). Values are shown as mean/hour ± SEM. N = 8–9. Grey areas in B, E, and F indicate the dark periods.

Many cancer patients treated with chemotherapeutics can develop CRF [4,9]. One class of chemotherapeutics, which is commonly used as single- or combination therapy for various types of tumors, are the topoisomerase inhibitors or poisons [11,12]. Doxorubicin, one of the most used and best-known family members, has been described to induce CRF in patients [8,29]. To explore the basis of this devastating side effect, we studied the effect of three different topoisomerase drugs (doxorubicin, aclarubicin, and etoposide) on the development of fatigue-like symptoms in healthy non-tumor-bearing mice. For this purpose, 12-week-old male C57BL/6J mice were subdivided into four groups, and baseline wheel-running activity was monitored (Figure 1A,B). Subsequently, mice were treated with four intra-peritoneal injections of the indicated cytostatic drugs over a period of 16 days. Treatment was followed by monitoring body weight (which is a representative parameter of general toxicity [30]), wheel-running activity, and cage activity under a normal 12 h:12 h light–dark (LD) cycle and in constant dark (DD) conditions to evaluate the effect on the circadian clock (Figure 1A,B and Supplementary Figure S1).

Mice treated with doxorubicin showed a decreased body mass compared with etoposide-, aclarubicin-, and control-treated mice. The weight loss started 9 days after the first injection (Figure 1C). Total body mass of doxorubicin-treated mice 48 days after the last injection was still 8% lower compared to their weight pre-treatment (Figure 1C). To analyze the treatment-induced fatigue-like symptoms, we quantified the wheel-running activity profile under LD and DD conditions (Figure 1B,E,F). Particularly, doxorubicin-treated mice showed decreased running both under LD and DD conditions compared to all other treatments and relative to their own baseline activity (Figure 1D). The 21-day total average running distance of doxorubicin-treated mice (101.45 ± 25.80 km) was markedly shorter than the running distance of aclarubicin (183.61 ± 28.95 km), etoposide (198.58 ± 16.78 km), and control (170.26 ± 17.63 km) treated mice (Figure 1D). Remarkably, when we quantified the 24-h cage locomotor activity (by passive infrared, PIR recording) profile over 10 days at baseline, under LD and DD conditions (Figure 1E), no significant effect of treatment was observed under LD conditions. In constant darkness, a small but significant decrease was observed in doxorubicin-treated mice (Figure 1E). Similar to the PIR recordings, no difference was observed for aclarubicin- or etoposide-treated mice when the 24-h wheel-running activity profile was analyzed (Figure 1F). On the other hand, doxorubicin-treated mice did show a significant decrease in 24-h wheel-running activity after treatment, both under LD and DD conditions, specifically in the first half of the night (Figure 1F). Overall, the changes in wheel-running activity following the different treatments indicate that doxorubicin, unlike etoposide or aclarubicin, induces fatigue-like symptoms in non-tumor-bearing mice. This suggests that the fatigue-like symptoms measured are determined by the type of cancer treatment.

### 3.2. Anthracycline Variants Have Diverse Underlying Mechanism of Action

**Figure 2:**
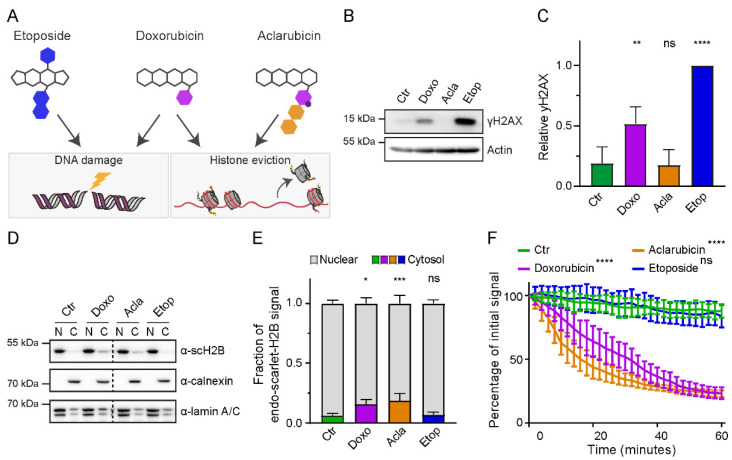
Mechanism of action of anthracycline drugs. (**A**) Schematic overview of the DNA damage (via targeting topoisomerase II) and chromatin damage (via eviction of histones) activity of the three anthracycline drugs used in this study. (**B**) Endogenously tagged scarlet-H2B U2Os cells were treated for 2 h with 10 µM of the indicated drugs. γH2AX levels were examined by Western blot, and actin was used as a loading control. (**C**) Quantification of the γH2AX levels normalized to actin. Ordinary one-way ANOVA with multiple comparisons; ns—not significant; ** *p* = 0.0052; **** *p* < 0.0001. (**D**) Endogenously tagged scarlet-H2B U2Os cells were treated for 1 h with 10 µM of the indicated drugs. Cells were fractionated, and the nuclear versus cytosolic fraction of H2B was examined by Western blot. Calnexin was used as cytosolic marker, and lamin A/C was used as nuclear marker. (**E**). Quantification of the nuclear versus cytosolic H2B signal from four independent experiments is plotted. Two-way ANOVA with multiple comparisons; ns—not significant; * *p* = 0.0109; *** *p* = 0.0007. (**F**). Quantification of the release of fluorescent PAGFP-H2A from the photoactivated nuclear regions upon administration of the indicated drugs. Ordinary one-way ANOVA with multiple comparisons; ns—not significant; **** *p* < 0.0001.

The anthracycline doxorubicin is associated with the induction of CRF [6]. This widely used chemotherapeutic is a topoisomerase II poison that functions by induction of DNA double-strand breaks following intercalation into the DNA and trapping of the enzyme [10,11]. In addition, doxorubicin also induces chromatin damage via eviction of histones (Figure 2A) [13,14]. To assess which activity (DNA damage or chromatin damage) is associated with the development of therapy-induced CRF, we tested two variant topoisomerase inhibitors: the anthracycline aclarubicin and the podophyllotoxin derivative etoposide. Similar to doxorubicin, etoposide also induces DNA double-strand breaks, as illustrated by the formation of γH2AX as detected by Western blot (Figure 2B,C) and fluorescent microscopy analysis (Supplementary Figure S2A,B). On the other hand, both doxorubicin and aclarubicin have chromatin damage activity (Figure 2D–F). To detect histone eviction, the cytosolic and nuclear fractions of cells treated with the different drugs were isolated, and endogenously tagged scarlet-H2B histones were detected by Western blot (Figure 2D,E). To confirm this observation, the localization of scarlet-H2B histones was analyzed by confocal microscopy (Supplementary Figure S2C,D). Alternatively, the fluorescent signal of photo-activated GFP-H2A histones was followed in living cells over time upon treatment with the different drugs (Figure 2F and Supplementary Figure S2E). These data confirm that both anthracyclines doxorubicin and aclarubicin, unlike etoposide, induced histone eviction. The difference in the underlying mechanism of action of these three cancer drugs inducing only DNA damage, only chromatin damage, or the combination of both, allows analyses of the contribution of these two biological effects within doxorubicin on CRF.

### 3.3. Sleep–Wake Patterns Were Not Affected by Chemotherapy Treatment

**Figure 3:**
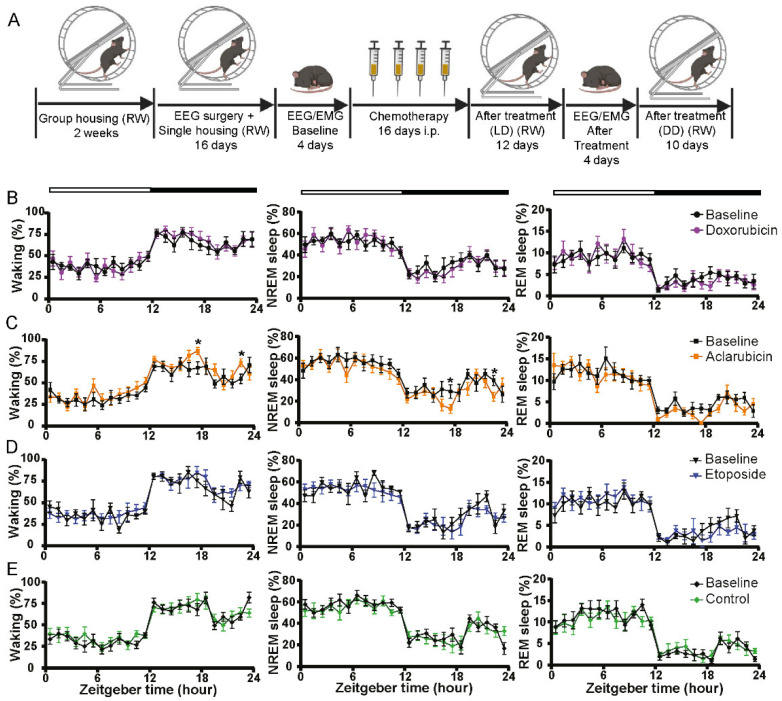
Chemotherapy-induced fatigue is not caused by changes in the sleep-wake rhythm. (**A**) Experimental design of the sleep–wake study. EEG—electroencephalogram, EMG—electromyography, RW—running wheel, LD—light-dark, DD—constant dark. (**B**–**E**) One-hour values of Waking, NREM, and REM sleep of 24 h before treatment baseline day compared with doxorubicin (**B**), aclarubicin (**C**), etoposide (**D**), and control (**E**) treatment. Values are shown as mean ± SEM. control n = 9, doxorubicin n = 8, aclarubicin n = 8, etoposide n = 7. Two-way ANOVA with Bonferroni multiple comparisons, compared with time-matched control values; * *p* < 0.05. White and black bars at the top indicate the light–dark cycle.

CRF has often been investigated in the framework of an increased need for sleep. Indeed, sleep problems are commonly reported by cancer patients undergoing chemotherapy [31,32]. These sleeping problems may, however, be independent of fatigue [33]. We, therefore, assessed the sleep–wake behavior in mice treated with either doxorubicin, etoposide, or aclarubicin. To do so, we performed EEG/electromyogram (EMG) recordings in freely moving mice with implanted stationary electrodes [25,27] before and after the completion of drug treatment (Figure 3A). Surprisingly, no significant differences in sleep and waking were observed for chemotherapeutically treated mice compared to baseline control or between treatment groups (Figure 3B–E). To further investigate the consequences of drug treatment in the different groups, we analyzed the EEG power density spectrum in the three vigilance states (Supplementary Figure S3). No alterations in the power density spectra were observed in either doxorubicin- or aclarubicin-treated mice compared to the baseline condition (Supplementary Figure S3A–F), whereas etoposide-treated mice exhibited increased EEG activity in the whole spectral range analyzed (0.5–25 Hz) in all three vigilance states (Supplementary Figure S3G–I). Since no relation to one particular vigilance state was observed, this suggests that etoposide induces a general overall change in brain activity. However, this general change in brain activity is not translated into an effect on fatigue symptoms, as no decline in wheel-running activity was observed in etoposide-treated mice (Figure 1). Taken together, the sleep and EEG data suggest that the reduction in voluntary wheel-running activity related to doxorubicin treatment is unrelated to an enhanced sleep need in these animals.

### 3.4. Doxorubicin-Treated Mice Exhibit a Disrupted Circadian Clock

**Figure 4:**
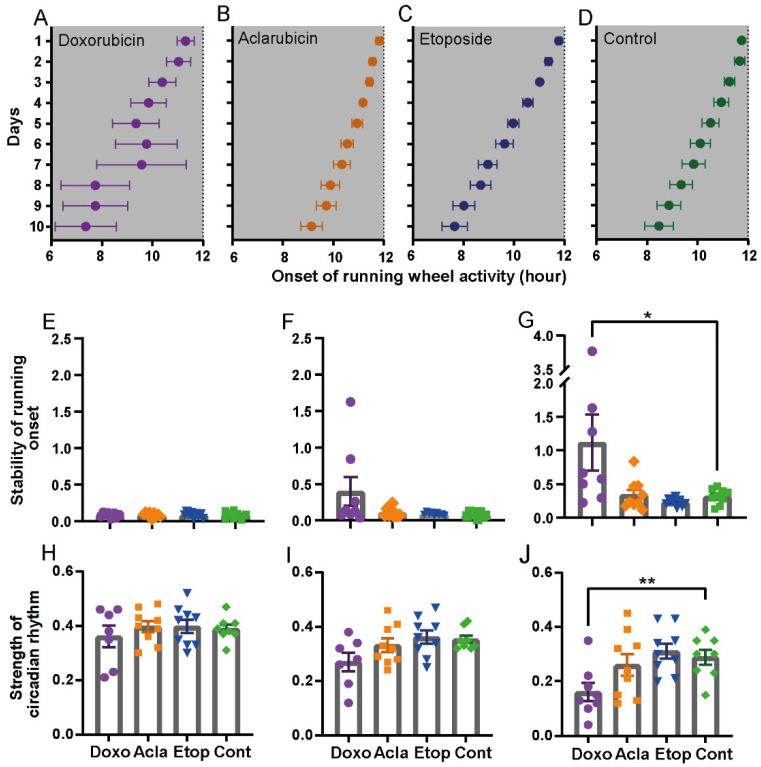
Doxorubicin-treated mice show a disrupted circadian clock. (**A**–**D**) Average wheel-running activity onset time of doxorubicin (**A**), aclarubicin (**B**), etoposide (**C**), and control (**D**) treated mice over a period of 10 days. Grey areas indicate the period of darkness. (**E**,**F**). Average 10 days stability of running onset time under baseline condition (**E**), treatment LD condition (**F**), and treatment DD condition (**G**). (**H**–**J)** Strength of the circadian rhythm (Qp) of wheel-running activity under baseline condition (**H**), treatment LD condition (**I**), and treatment DD condition (**J**). Data are shown as mean ± SEM. doxorubicin n = 8, all other treatments n = 9 per group. One-way ANOVA with Bonferroni multiple comparison; * *p* < 0.05, ** *p* < 0.01.

If fatigue is not related to enhanced sleep and/or sleep pressure, we considered an altered waking state quality as an underlying cause rather than a change in sleep quality. In humans, wakefulness during the day is promoted by the circadian clock of the suprachiasmatic nucleus [34]. So far, only one study has investigated the possible relation between therapy-induced CRF and a disrupted circadian clock [24]. To investigate whether doxorubicin-induced CRF might be a consequence of a disrupted circadian clock, we analyzed the precision of the behavioral rest-activity rhythm by measuring the daily onset of activity of the different treatment groups (Supplementary Figure S4), which is a routine way to test the expression of the endogenous clock [35]. Under DD conditions, doxorubicin-treated mice exhibited an unstable activity onset compared to all other groups (Figure 4A–D), while under baseline- and LD conditions following treatment, no difference between the three drugs and control groups was observed (Supplementary Figure S4B,E,H,K). To quantify this finding, we calculated the variability in activity onset over 10 days for the individual mice. Under baseline and treated LD conditions, the differences were not significant (Figure 4E,F), whereas under DD, the doxorubicin-treated mice showed vastly unstable activity onsets compared to the other treatments (Figure 4G). This suggests that treatment with doxorubicin destabilizes the circadian clock and abolishes the ability to properly time day-to-day behavior in the absence of external time cues (Figure 4A–D,G). Subsequently, we determined the period and strength of the circadian rhythm (Qp) by F-periodogram analyses [28] of mice treated with the different drugs at baseline and in LD or DD conditions (Supplementary Figure S1C). The period did not differ between the four treatment groups (doxorubicin 23.54 ± 0.14, aclarubicin 23.78 ± 0.05, etoposide 23.55 ± 0.05, saline control 23.69 ± 0.06, *p* = 0.1061, mean ± SEM, one-way ANOVA). Qp was significantly lower after doxorubicin treatment under DD conditions compared to control mice, unlike mice in the other groups (Figure 4H–J).

Overall, the data show that the present mouse model of CRF can discriminate between different working mechanisms of cancer treatment drugs. In addition, sleep is not affected by treatment with the drug that causes fatigue. In contrast, therapy-induced CRF is likely caused by alterations in the central circadian pacemaker as circadian rest-activity behavior shows less precise timing and has a reduced rhythm strength in mice treated with doxorubicin.

## 4. Discussion

Cancer-treatment-related fatigue following chemo- or radiotherapy is a devastating side effect and complaint of many cancer survivors [4]. This adverse effect accumulates during therapy and can last for a long and undefined period of time [31]. Persistent CRF has a serious and continuing effect on the quality of life of these patients since they are too tired to work, socialize, or even perform normal daily activities [1,3]. Despite the high incidence of CRF, its cause is poorly understood. Furthermore, without a clear definition of fatigue, unambiguity for measuring fatigue symptoms, and a (golden standard) therapy for CRF, the study of fatigue is complex [5]. Consequently, most studies related to the evaluation of CRF concentrate on psychological assessment and treatment [36]. To better understand the underlying molecular mechanisms of chemotherapy-induced CRF, we decided to study the effects of three topoisomerase II targeting drugs (doxorubicin, etoposide, and aclarubicin), commonly used in cancer treatment, on CRF-like symptoms in tumor-free mice [11,12]. Although these three variants belong to the same family of chemotherapeutic drugs, they function through different mechanisms. Where etoposide induces DNA double-strand breaks, aclarubicin is abstained from DNA damage activity but induces chromatin damage via eviction of histones [13]. In the case of doxorubicin, both activities (DNA- and chromatin damage) are combined in one molecule. For this reason, we wondered whether the combination of activities within doxorubicin would also be essential for the development of therapy-induced CRF in patients treated with this class of chemotherapeutic drugs.

Our current study shows that there is a clear difference in fatigue symptoms and severity between doxorubicin and its family members. Where doxorubicin treatment resulted in decreased voluntary running, both under LD and DD conditions, none of the other treatments showed an effect different from baseline activity. Furthermore, the 21-day total running distance was remarkably lower in doxorubicin-treated mice compared to other groups. These data do not prove that the combination of the two working mechanisms causes the fatigue-like symptoms, as it may also be related to other types of toxicity, but they do indicate that treatment-related CRF is therapy-specific and, in the present study, is only correlated with the topoisomerase drug that combines both DNA- and chromatin damage activity. The observed effect on fatigue symptoms by doxorubicin could be explained by attenuated DNA repair as a consequence of both DNA damage induction and eviction of H2AX histones, which are then no longer available for phosphorylation, as is observed for other toxicities associated with doxorubicin treatment [12].

Moreover, doxorubicin caused fatigue-like behavior, with a clear influence on voluntary wheel-running activity, but it had hardly any effect on levels of other (locomotor) activity within the cage, recorded with passive infrared sensors, which is much less intense and includes foraging, eating and drinking. Therefore, the treatment with doxorubicin seems to affect specifically voluntary wheel-running behavior but leaves other, less intense behavior unaffected. This observation is in line with a previous study on CRF in doxorubicin-treated mice [17] and confirms the value of our model and wheel-running activity as a good readout for fatigue.

Given the expected interaction between sleep need and CRF, we investigated sleep quantity and quality in the different treatment groups. Sleep was recorded three weeks after completion of treatment. Surprisingly, we did not observe any significant difference in the amount or distribution of the three vigilance states, despite the obvious difference in the wheel running activity levels. We, therefore, hypothesized that therapy-related CRF might be caused by a disrupted regulation of the active period by the central circadian clock instead of being a problem related to sleep. This hypothesis is supported by our analysis of the wheel-running recordings performed under DD conditions, which show that both the precision and the strength of the circadian rhythm were reduced in doxorubicin-treated mice. The different treatments did not seem to affect the endogenous circadian period of the circadian clock but rather seemed to influence the precision of timing of onset of activity. Intriguingly, a recent study on the effect of paclitaxel, a commonly used taxane chemotherapeutic, on CRF in mice also shows disruption of the circadian clock [24]. However, paclitaxel treatment only results in a temporary decrease in wheel-running activity, which is different from our doxorubicin-treated mice with persistent CRF symptoms. This discrepancy could be explained by the difference in the molecular working mechanisms of these chemotherapeutics. Where paclitaxel is known to induce transient DNA damage by arresting cell growth by microtubule stabilization [37], doxorubicin treatment is known to attenuate DNA damage repair upon induction of DNA breaks due to the eviction of yH2AX marked histones (Figure 2) [13]. This might then affect the extent and duration of fatigue-like symptoms induced by doxorubicin. Our data showing persistent fatigue symptoms induced by doxorubicin treatment (symptoms last until the end of the experiment, several weeks after treatment) are in line with observations of chronic CRF made in patients [3,38]. Our findings indicate that doxorubicin has a long-lasting effect on circadian-regulated rest-activity behavior, unlike etoposide or aclarubicin. Therapy-related CRF is thus strongly dependent on the type of treatment.

In the present study, we decided to include only male animals, as previous studies in male mice had provided a reasonable estimate of the dosage to use and the methods to apply [15,17]. To rule out any gender difference in the induction of therapy-induced CRF, future studies of the difference between males and females would be highly interesting to study the putative variation in response depending on sex. Furthermore, investigating where in the processing of circadian rhythms the treatments are most disturbing additional measures on circadian rhythms, for instance, body temperature, metabolism changes, and metabolomics, could improve our understanding of the mechanism of therapy-induced CRF.

In mammals, the rest–activity cycle is under the control of the central circadian pacemaker located in the suprachiasmatic nucleus (SCN) of the hypothalamus [39]. The SCN influences activity in brain areas such as the lateral hypothalamus, prefrontal cortex, and nucleus accumbens, which controls voluntary activity, arousal, and wakefulness, among others [40,41]. A chronic effect of chemotherapy on the central clock may therefore result in a decreased possibility to express activity during the day. Our observation of doxorubicin-induced fatigue is consistent with clinical studies showing that patients treated with chemotherapy have a dampened day-night rhythm compared to healthy volunteers [42].

## 5. Conclusions

Our data show that treatment with doxorubicin, which combines both DNA- and chromatin damage activity, results in increased fatigue symptoms in tumor-free mice. In contrast, treatment with aclarubicin or etoposide failed to induce fatigue, suggesting that the development of this devastating side effect is probably the result of specific cancer treatment mechanisms. These symptoms are not associated with changes in sleep but rather with a disrupted circadian clock. CRF is then dependent on the type of chemotherapy, which could be an important criterium for selecting cancer drugs for treatment.

## Data Availability

The data generated in this study are available upon request from the corresponding author.

## Supplementary Materials

The following supporting information can be downloaded at: https://www.mdpi.com/article/10.3390/cancers14102421/s1, Figure S1: Schematic representation of wheel running and passive infrared recording (PIR). Figure S2: Mechanism of action of anthracycline drugs. Figure S3: Electroencephalographic (EEG) power density in waking, NREM, and REM sleep of four groups under baseline and treatment conditions. Figure S4: Onset of wheel-running activity of four groups.

## References

[1] Cella D., Davis K., Breitbart W., Curt G., Coalition F.T.F. (2001). Cancer-Related Fatigue: Prevalence of Proposed Diagnostic Criteria in a United States Sample of Cancer Survivors. J. Clin. Oncol..

[2] Hofman M., Ryan J.L., Figueroa-Moseley C.D., Jean-Pierre P., Morrow G.R. (2007). Cancer-Related Fatigue: The Scale of the Problem. Oncol..

[3] Servaes P., Gielissen M.F.M., Verhagen S., Bleijenberg G. (2006). The course of severe fatigue in disease-free breast cancer patients: A longitudinal study. Psycho Oncol..

[4] Schwartz A.L., Nail L.M., Chen R.N., Meek P., Barsevick A.M., King M.E., Jones L.S. (2000). Fatique Patterns Observed in Patients Receiving Chemotherapy and Radiotherapy. Cancer Investig..

[5] Stone P., Richardson A., Ream E., Smith A.G., Kerr D.J., Kearney N. (2000). Cancer-related fatigue: Inevitable, unimportant and untreatable? Results of a multi-centre patient survey. Ann. Oncol..

[6] Yang S., Chu S., Gao Y., Ai Q., Liu Y., Li X., Chen N. (2019). A Narrative Review of Cancer-Related Fatigue (CRF) and Its Possible Pathogenesis. Cells.

[7] Bower J.E., Lamkin D.M. (2012). Inflammation and cancer-related fatigue: Mechanisms, contributing factors, and treatment implications. Brain Behav. Immun..

[8] Prigozin A., Uziely B., Musgrave C.F. (2010). The Relationship Between Symptom Severity and Symptom Interference, Education, Age, Marital Status, and Type of Chemotherapy Treatment in Israeli Women with Early-Stage Breast Cancer. Oncol. Nurs. Forum.

[9] Bower J.E. (2014). Cancer-related fatigue—mechanisms, risk factors, and treatments. Nat. Rev. Clin. Oncol..

[10] Tewey K.M., Rowe T.C., Yang L., Halligan B.D., Liu L.F. (1984). Adriamycin-Induced DNA Damage Mediated by Mammalian DNA Topoisomerase II. Science.

[11] Nitiss J.L. (2009). Targeting DNA topoisomerase II in cancer chemotherapy. Nat. Rev. Cancer.

[12] Van der Zanden S.Y., Qiao X., Neefjes J. (2020). New insights into the activities and toxicities of the old anticancer drug doxorubicin. FEBS J..

[13] Pang B., Qiao X., Janssen L., Velds A., Groothuis T., Kerkhoven R., Nieuwland M., Ovaa H., Rottenberg S., van Tellingen O. (2013). Drug-induced histone eviction from open chromatin contributes to the chemotherapeutic effects of doxorubicin. Nat. Commun..

[14] Yang F., Kemp C.J., Henikoff S. (2013). Doxorubicin Enhances Nucleosome Turnover around Promoters. Curr. Biol..

[15] Qiao X., van der Zanden S.Y., Wander D.P.A., Borràs D.M., Song J.-Y., Li X., van Duikeren S., van Gils N., Rutten A., van Herwaarden T. (2020). Uncoupling DNA damage from chromatin damage to detoxify doxorubicin. Proc. Natl. Acad. Sci. USA.

[16] Wander D.P.A., van der Zanden S.Y., van der Marel G.A., Overkleeft H.S., Neefjes J., Codée J.D.C. (2020). Doxorubicin and Aclarubicin: Shuffling Anthracycline Glycans for Improved Anticancer Agents. J. Med. Chem..

[17] Zombeck J.A., Fey E.G., Lyng G.D., Sonis S.T. (2013). A clinically translatable mouse model for chemotherapy-related fatigue. Comp. Med..

[18] Borniger J.C., Gaudier-Diaz M.M., Zhang N., Nelson R.J., DeVries A.C. (2014). Cytotoxic chemotherapy increases sleep and sleep fragmentation in non-tumor-bearing mice. Brain Behav. Immun..

[19] Meijer J.H., Robbers Y. (2014). Wheel running in the wild. Proc. R. Soc. B Boil. Sci..

[20] Gu C., Coomans C.P., Hu K., Scheer F.A.J.L., Stanley H.E., Meijer J.H. (2015). Lack of exercise leads to significant and reversible loss of scale invariance in both aged and young mice. Proc. Natl. Acad. Sci. USA.

[21] Sullivan K.A., Grant C.V., Jordan K.R., Vickery S.S., Pyter L.M. (2020). Voluntary wheel running ameliorates select paclitaxel chemotherapy-induced sickness behaviors and associated melanocortin signaling. Behav. Brain Res..

[22] Ray M.A., Trammell R.A., Verhulst S., Ran S., Toth L.A. (2011). Development of a mouse model for assessing fatigue during chemotherapy. Comp. Med..

[23] Wood L.J., Nail L.M., Perrin N.A., Elsea C.R., Fischer A., Druker B.J. (2006). The Cancer Chemotherapy Drug Etoposide (VP-16) Induces Proinflammatory Cytokine Production and Sickness Behavior–like Symptoms in a Mouse Model of Cancer Chemotherapy–Related Symptoms. Biol. Res. Nurs..

[24] Sullivan K.A., Grant C.V., Jordan K.R., Obrietan K., Pyter L.M. (2021). Paclitaxel chemotherapy disrupts behavioral and molecular circadian clocks in mice. Brain Behav. Immun..

[25] Panagiotou M., Papagiannopoulos K., Rohling J.H.T., Meijer J.H., DeBoer T. (2018). How Old Is Your Brain? Slow-Wave Activity in Non-rapid-eye-movement Sleep as a Marker of Brain Rejuvenation After Long-Term Exercise in Mice. Front. Aging Neurosci..

[26] De Boer T., Ruijgrok G., Meijer J.H. (2007). Short light-dark cycles affect sleep in mice. Eur. J. Neurosci..

[27] Panagiotou M., Vyazovskiy V.V., Meijer J.H., DeBoer T. (2017). Differences in electroencephalographic non-rapid-eye movement sleep slow-wave characteristics between young and old mice. Sci. Rep..

[28] Stenvers D.J., Van Dorp R., Foppen E., Mendoza J., Opperhuizen A.-L., Fliers E., Bisschop P.H., Meijer J.H., Kalsbeek A., Deboer T. (2016). Dim light at night disturbs the daily sleep-wake cycle in the rat. Sci. Rep..

[29] De Jong N., Candel M.J.J.M., Schouten H.C., Abu-Saad H.H., Courtens A.M. (2004). Prevalence and course of fatigue in breast cancer patients receiving adjuvant chemotherapy. Ann. Oncol..

[30] Gabizon A., Meshorer A., Barenholz Y. (1986). Comparative long-term study of the toxicities of free and liposome-associated dox-orubicin in mice after intravenous administration. J. Natl. Cancer Inst..

[31] Bower J.E., Ganz P.A., Desmond K.A., Rowland J.H., Meyerowitz B.E., Belin T.R. (2000). Fatigue in Breast Cancer Survivors: Occurrence, Correlates, and Impact on Quality of Life. J. Clin. Oncol..

[32] Liu L., Mills P.J., Rissling M., Fiorentino L., Natarajan L., Dimsdale J.E., Sadler G.R., Parker B.A., Ancoli-Israel S. (2012). Fatigue and sleep quality are associated with changes in inflammatory markers in breast cancer patients undergoing chemotherapy. Brain Behav. Immun..

[33] Ancoli-Israel S., Moore P.J., Jones V. (2001). The relationship between fatigue and sleep in cancer patients: A review. Eur. J. Cancer Care.

[34] Dijk D., Czeisler C. (1995). Contribution of the circadian pacemaker and the sleep homeostat to sleep propensity, sleep structure, electroencephalographic slow waves, and sleep spindle activity in humans. J. Neurosci..

[35] Welsh D.K., Engle E.M.R.A., Richardson G.S., Dement W.C. (1986). Precision of circadian wake and activity onset timing in the mouse. J. Comp. Physiol. A Sens. Neural Behav. Physiol..

[36] Kangas M., Bovbjerg D.H., Montgomery G.H. (2008). Cancer-related fatigue: A systematic and meta-analytic review of non-pharmacological therapies for cancer patients. Psychol. Bull..

[37] Branham M.T., Nadin S., Vargas-Roig L.M., Ciocca D.R. (2004). DNA damage induced by paclitaxel and DNA repair capability of peripheral blood lymphocytes as evaluated by the alkaline comet assay. Mutat. Res. Toxicol. Environ. Mutagen..

[38] Bower J.E., Ganz P.A., Desmond K.A., Bernaards C., Rowland J.H., Meyerowitz B.E., Belin T.R. (2006). Fatigue in long-term breast carcinoma survivors. Cancer.

[39] Meijer J.H., Rietveld W.J. (1989). Neurophysiology of the suprachiasmatic circadian pacemaker in rodents. Physiol. Rev..

[40] Basso J.C., Morrell J.I. (2015). The medial prefrontal cortex and nucleus accumbens mediate the motivation for voluntary wheel running in the rat. Behav. Neurosci..

[41] Dreher J.K., Jackson D.M. (1989). Role of D1 and D2 dopamine receptors in mediating locomotor activity elicited from the nucleus accumbens of rats. Brain Res..

[42] Rich T.A. (2007). Symptom clusters in cancer patients and their relation to EGFR ligand modulation of the circadian axis. J. Support. Oncol..

