# Supplementary material for: Induction of Fatigue by Specific Anthracycline Cancer Drugs through Disruption of the Circadian Pacemaker
Source: Cancers (Basel). 2022 May 13;14(10):2421. doi: 10.3390/cancers14102421 (PMC9140011; doi:10.3390/cancers14102421)
Supplement: Supplementary file 1 [file cancers-14-02421-s001.zip › cancers-1703687-supplementary.pdf]

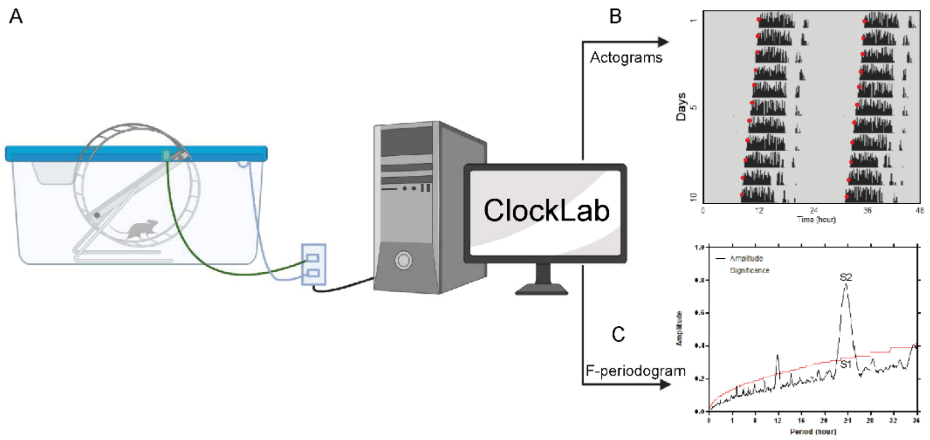

Supplementary Figure S1. Schematic representation of wheel running and passive infrared recording (PIR). Wheel running and PIR data were recorded in the Clocklab system (A). The onset of the behavioral activity was shown in the double plotted actograms with red dot (B). The period and strength of circadian clock (Qp) were determined from the F-periodogram (C). The strength of the rhythms was determined as the difference between the peak (s2) and the corresponding 95% confidence limit (s1) in F-periodogram, as previously described [30,34].

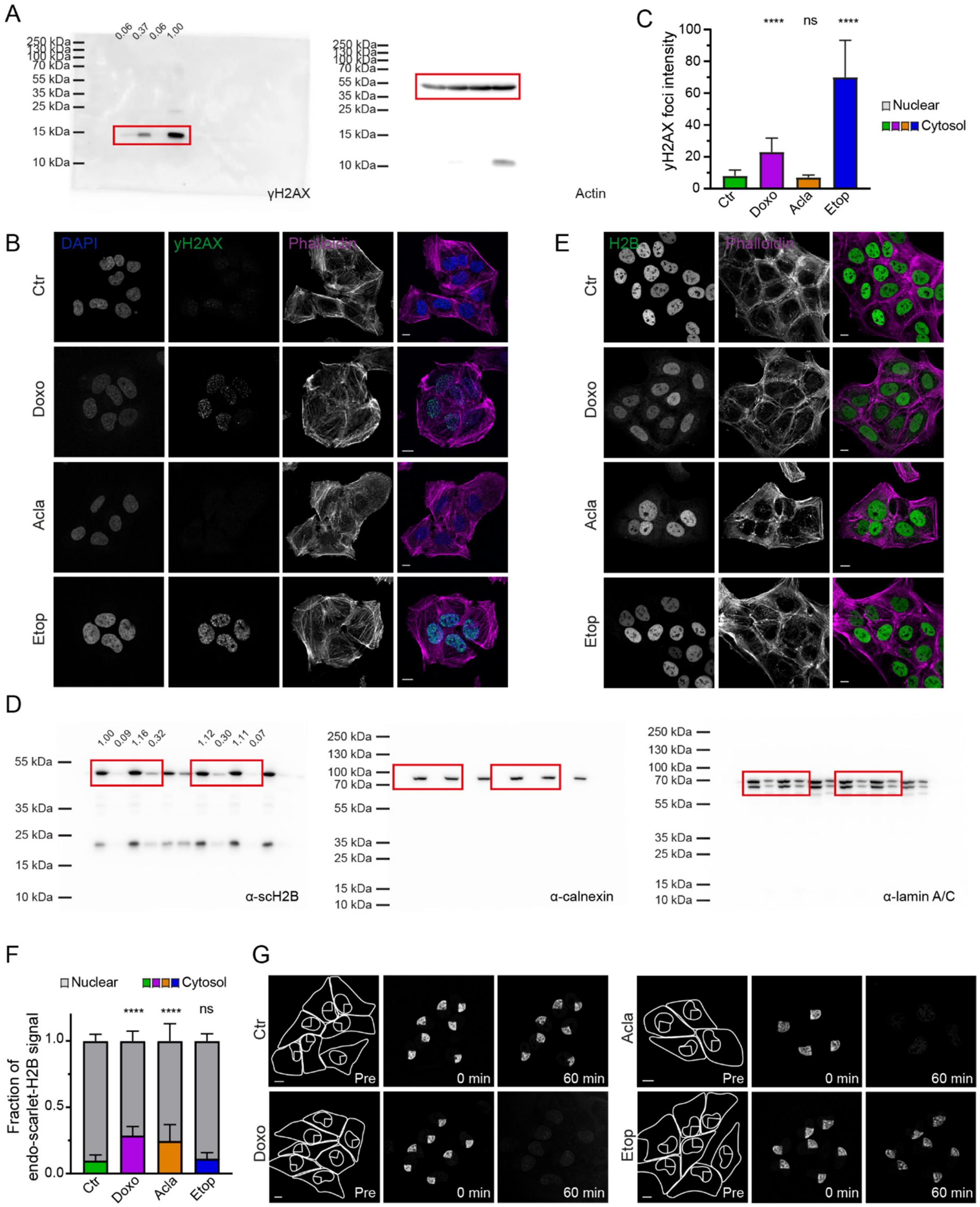

Supplementary Figure S2. Mechanism of action of anthracycline drugs. A. Whole Western blot corresponding to Fig. 2B. Cut out bands are indicated by the red box. Densitometry intensity ratio is indicated above the band. B. γH2AX (green) foci formation upon anthracycline treatment. Representative confocal image of fixed U2Os cells treated for 1 hour with the indicated drugs. DAPI (blue) and Phalloidin (magenta) are stained as nuclear and cytosol marker, respectively. Scale bar; 10 μm. C. Quantification of the γH2AX foci induced upon treatment, nCtr = 28, nDoxo = 37, nAcla = 34, nEtop = 35 analyzed from two independent experiments. Two-way ANOVA with multiple comparisons; ns, not significant, P **** < 0.0001.

D. Whole Western blot corresponding to Fig. 2D. Cut out bands are indicated by the red box. Densitometry intensity ratio is indicated above the bands. E. Nuclear versus cytosolic localization of endogenous H2B levels upon 1 hour treatment with 10µM of the indicated drugs. Representative confocal image of fixed endogenously tagged scarlet-H2B U2Os cells. Scale bar, 10 µm. F. Quantification of the nuclear versus cytosolic H2B signal, nCtr = 105, nDoxo = 102, nAcla = 106, nEtop = 105 analyzed from two independent experiments is plotted. Two-way ANOVA with multiple comparisons; ns, not significant, P **** < 0.0001. G. Part of the nucleus from MelJuSo cells stably expressing PAGFP-H2A was photo-activated. Photo-activated PAGFP-H2A was monitored by time-lapse confocal microscopy for 1 hour in the absence or presence of the indicated drug at 10 µM. Lines in the left panel define the region of cytoplasm, nucleus and activated area pre-treatment. Stills from a movie at 0 and 60 minutes after addition of the drugs are shown. Scale bar; 10 µm.

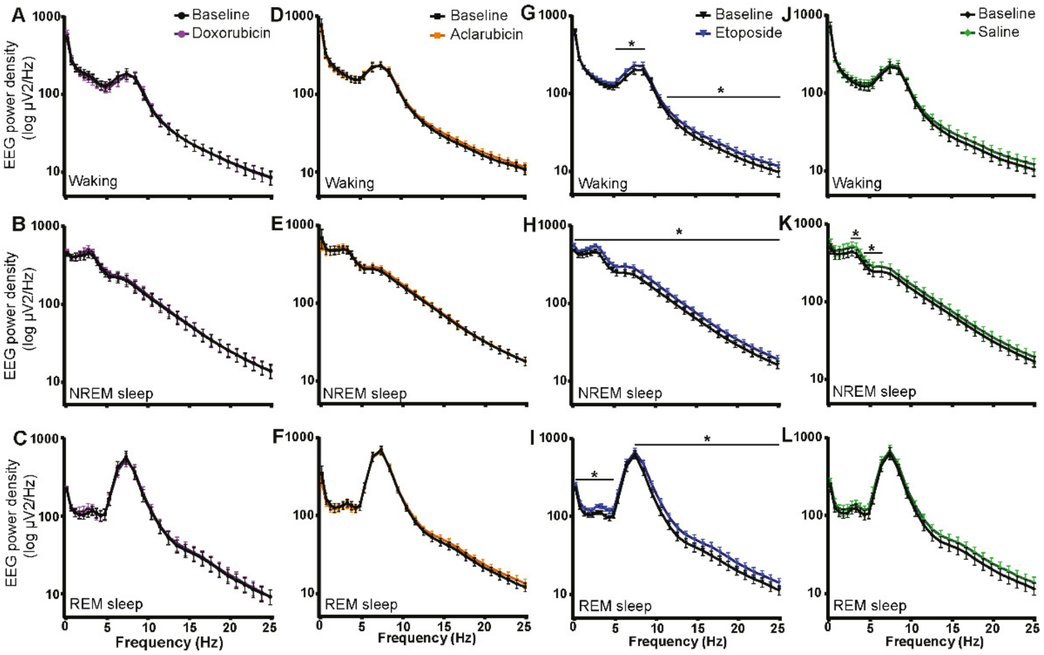

Supplementary Figure S3. Electroencephalographic (EEG) power density in Waking, NREM and REM sleep of four groups under baseline and treatment condition. Spectra are shown of Waking (A, D, G, J), NREM sleep (B, E, H, K) and REM sleep (G, F, I, L) EEG for the 24-hour before treatment baseline day and three weeks after completion of chemotherapy treatment. Power density values were calculated in 0.5-Hz bins between 0.25 and 5.0 Hz, 1-Hz bins were calculated between 5.25 and 25.0 Hz. P * = 0.05-0.0001 compared with frequency-matched baseline values, two-way ANOVA with Bonferroni multiple comparisons.

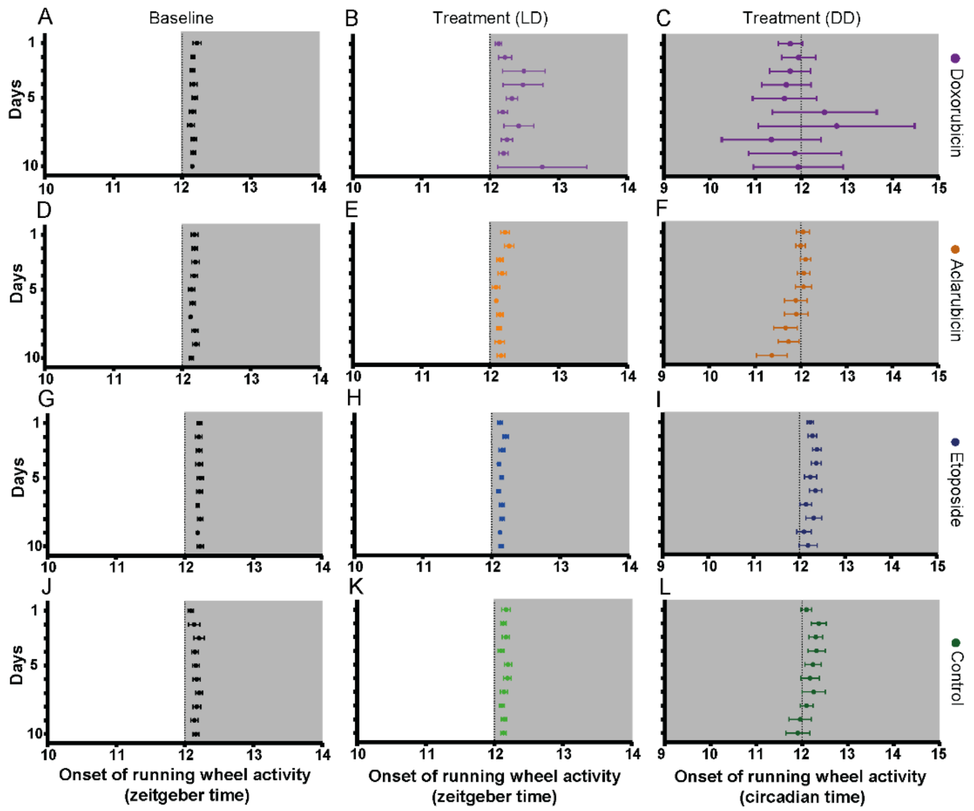

Supplementary Figure S4. Onset of wheel running activity of four groups. Average running activity onset time of doxorubicin, aclarubicin, etoposide and control treated mice over 10 days under baseline condition (A, D, G, J), treatment LD condition (B, E, H, K) and treatment DD condition (C, F, I, L). Grey areas indicate the period of darkness.
